# Genetic analysis using long-read sequencing to overcome the difficulties in *VWF* gene

**DOI:** 10.1016/j.rpth.2025.102888

**Published:** 2025-05-17

**Authors:** Sheng Ye, Yuka Eura, Masanori Matsumoto, Koichi Kokame

**Affiliations:** 1 Department of Molecular Pathogenesis, National Cerebral and Cardiovascular Center, Suita, Osaka, Japan; 2 Department of Cardiovascular System Research, Nara Medical University, Kashihara, Nara, Japan; 3 Department of Blood Transfusion Medicine and Hematology, Nara Medical University, Kashihara, Nara, Japan

**Keywords:** von Willebrand factor, von Willebrand disease, nanopore sequencing, polymerase chain reaction

## Abstract

**Background:**

Genetic defects in von Willebrand factor (VWF) can lead to von Willebrand disease (VWD). Identifying causative or modifier variants of *VWF* is crucial for the diagnosis, classification, and clinical management of VWF disorders. However, owing to the length (178 kb) and complexity of *VWF* and the presence of the pseudogene *VWFP1*, Sanger sequencing or short-read next-generation sequencing is often challenging.

**Objectives:**

This study aimed to establish a long-read sequencing method using Oxford nanopore technology (ONT) to overcome difficulties associated with *VWF* gene analysis.

**Methods:**

Genetic analyses were established using genomic DNA from a healthy donor and validated using 3 VWF disorder patient samples. Long-range (∼15 kb) polymerase chain reaction was optimized to obtain 21 amplicons covering the entire *VWF* gene, avoiding unwanted amplification due to repetitive sequences and *VWFP1*. ONT nanopore sequencing data were analyzed using software programs, including Clair3, Longshot, and Sniffles. The identified candidate variants were verified by several approaches such as Sanger sequencing and haplotyping.

**Results:**

The entire *VWF* gene was successfully read using ONT nanopore sequencing, with >200 variants called in each patient sample. A rare missense variant, p.(Gln2442His) and a rare 2599 bp deletion were identified in patients 2 and 3, respectively. However, the deletion was confirmed as long-range polymerase chain reaction artifacts, which warrant attention when using this method.

**Conclusion:**

This study presents an optimal solution using ONT nanopore sequencing to identify variants in *VWF*, which may improve the diagnosis of VWF disorders.

## Essentials

• von Willebrand factor (VWF) gene is large and complex, making genetic analysis challenging.
• This study used Oxford Nanopore Technology for long-read sequencing of the *VWF* gene.
• Oxford Nanopore Technology identified ∼200 variants per patient, including a rare missense mutation and a large deletion.
• Long-read sequencing may improve diagnosis of VWF-related disorders by detecting complex genetic changes.

## 1 Introduction

von Willebrand factor (VWF) is a multimeric plasma glycoprotein essential for normal hemostasis by promoting the platelet adhesion to subendothelial collagen exposed during vascular injury [[1], [2], [3]]. VWF is also important for protecting factor (F)VIII from proteolysis in the circulation [4]. High-molecular-weight multimers of VWF are considered to be crucial for its function in the plasma [5,6].

The VWF gene (*VWF*) is located on chromosome 12 (12p13.31), consists of 52 exons and is approximately 178 kb in length [4,7]. Abnormalities in the VWF protein due to *VWF* mutations would result in von Willebrand disease (VWD), a common inherited bleeding disorder with an estimated prevalence from 1 per 1000 to 1 per 100 population, while the real prevalence is still not well established yet [[8], [9], [10]]. VWD is classified into 3 main types [11,12], and in most patients, VWF antigen or binding affinity tests can readily differentiate the VWD type and help patients receive the most appropriate treatment [13]. Genetic analysis also plays an important role in providing a more precise diagnosis, thereby supporting the clinical management of VWD, particularly in patients with atypical presentations. For example, it enables differentiation between type 2N VWD and hemophilia A in males, distinguishes type 2B VWD from platelet-type VWD, and can be applied in prenatal diagnosis for families affected by type 3 VWD [14]. Furthermore, examining the relationship between *VWF* genetic variation and plasma VWF activity can contribute to prevalence estimation in specific populations, as exemplified by the Suita study, which investigated the frequency of hereditary thrombotic thrombocytopenic purpura in the Japanese population [15].

Nevertheless, the analysis of *VWF* is complicated by several factors in addition to its large size. For example, a number of repetitive sequences have been identified, including 14 Alu repeats and approximately 670-bp TCTA simple repeat in intron 40, which may be polymorphic [7]. Moreover, a 21- to 29-kb *VWFP1* pseudogene located on chromosome 22q11-13 shares 97% sequence identity with *VWF* exons 23 to 34 [16]. These characteristics further complicate specific polymerase chain reaction (PCR) amplification and DNA sequencing and pose notable obstacles to genetic analyses. Commonly used techniques, such as Sanger sequencing and short-read next-generation sequencing (NGS), frequently encounter difficulties in this context [17,18].

To address this issue, we used a long-read sequencing platform, Oxford nanopore technology (ONT). ONT is a combination of genetic engineering and computational technology that can directly analyze single DNA molecules in real time and is therefore commonly referred to as third-generation sequencing or single-molecule sequencing. ONT identifies different nucleotides by measuring changes in electrical charge as DNA passes through biological nanopores [19]. It can provide exceptionally long reads (10-100 kb) that allow direct sequencing of regions previously inaccessible or problematic for short-read platforms, including long repetitive elements, regions with extreme GC content, and complex gene loci [20]. With these prominent advantages of long-read lengths and fast sequencing times (2-10 hours), ONT has become one of the most powerful tools in genomic research, used not only for identifying small single-nucleotide variants (SNVs) but also for the application of direct haplotype phasing, detection of complex structural variants (SVs), de novo genome assembly, and telomere-to-telomere chromosome assemblies [[21], [22], [23], [24]].

**Figure 1:**
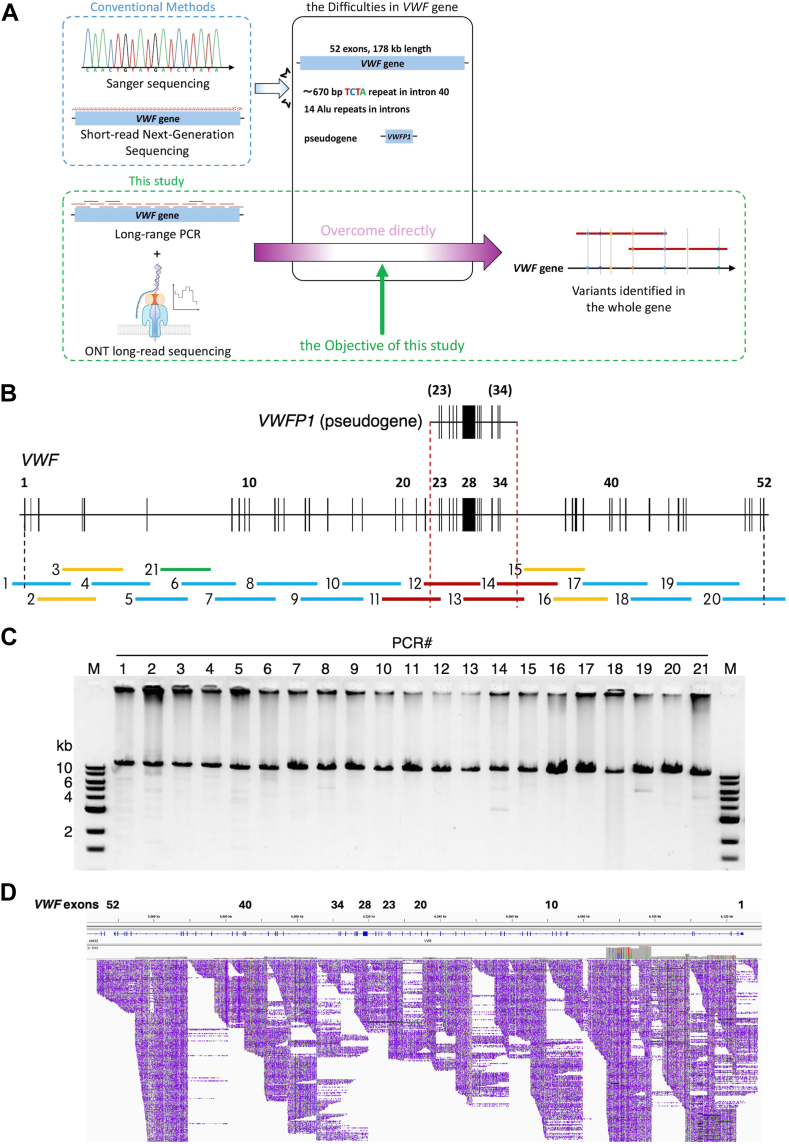
Long-read sequencing design and results. (A) Schematic representation of the advantages of long-read sequencing for *VWF* genetic analysis and the objective of this study. The red lines above the *VWF* gene indicate polymerase chain reaction (PCR) amplicons generated from DNA samples. (B) Design of long-range PCR covering the entire *VWF* gene. PCR2, PCR3, PCR15, and PCR16 (yellow) represent regions where amplification was unsuccessful with the initial primer pair. PCR11-PCR14 (red) target the pseudogene homology region, while PCR21 (green) spans a short overlapping region between PCR5 and PCR6. (C) Representative image of 1.2% agarose gel electrophoresis of PCR amplicons generated from a healthy donor DNA sample. M, DNA ladders of 1.5, 2, 3, 4, 5, 6, 8, and 10 kb. (D) ONT nanopore sequencing reads from healthy donor samples visualized using Integrative Genomics Viewer software. The numbers indicate the positions of exons 1, 10, 20, 23, 28, 34, 40, and 52. ONT, Oxford nanopore technology.

Although ONT technology is currently used for long-read whole-genome sequencing, its application remains limited by high costs, imprecise base-calling accuracy, and relatively low coverage in target gene regions [25]. To overcome these challenges and achieve sufficient read depth for more precise base calling in the genetic analysis of *VWF*, we developed an optimal workflow using long-range PCR and an ONT platform (Figure 1A). First, we demonstrated the efficacy of this method by performing genetic analyses on a healthy donor. The results confirmed that the entire *VWF* gene, including intronic regions, was successfully sequenced. We then validated its applicability in VWF-related disorders using DNA samples from 3 patients registered under the keyword “VWD” in the National Cerebral and Cardiovascular Center (NCVC) Biobank. Identified candidate variants were confirmed through methods such as Sanger sequencing and haplotyping.

To our knowledge, this is the first report describing the use of ONT nanopore sequencing in *VWF* research. This method has the potential for broad application in disease investigation and clinical management of VWF-related disorders in the near future.

## 2 Methods

### 2.1 DNA samples

Genomic DNA (gDNA) samples from the health donor and 3 patients were prepared from whole blood specimens using the Puregene Blood Kit and QIAsymphony (Qiagen). This study was approved by the NCVC Research Ethics Committee (approval number R22005-5).

### 2.2 Long-range PCR

The design of the long-range PCR for *VWF* is shown in Figure 1B. PCR primer pairs used to amplify 21 amplicons (12-15 kb) covering the entire *VWF* gene with a 3- to 8-kb overlap were designed using Primer-BLAST at National Institutes of Health with single-nucleotide polymorphism (SNP) handling, repeat and low complexity filters. For regions that could not be amplified by primer pairs with these strict requirements, each region was replaced by 2 overlapping regions that covered a longer distance and can be amplified using qualified primer pairs (Figure 1B, yellow). To amplify the 4 amplicons covering the pseudogene homology region (Figure 1B, red), 2 forward and 2 reverse primers were designed outside the homology region, and the corresponding reverse or forward primers were designed inside the region [16]. All primer sequences are listed in Supplementary Table 1.

Each PCR reaction mix consisted of 0.2 μM each of the forward and reverse primer, 1× PrimeSTAR GXL Buffer, 0.2 mM dNTP mix, 100 to 300 ng gDNA, and 1.25 units of PrimeSTAR GXL DNA Polymerase (Takara Bio), combined in a 50-μL reaction. The PCR cycling conditions were as follows: For PCR1 to PCR12 and PCR16 to PCR21, initial denaturation of 94 °C for 2 minutes, followed by 30 cycles of 98 °C for 10 seconds, 68 °C for 10 minutes, followed by a final extension of 68 °C for 5 minutes; for PCR13 to PCR15, initial denaturation of 94 °C for 2 minutes, followed by 30 cycles of 98 °C for 10 seconds, 60 °C for 15 seconds, 68 °C for 10 minutes, followed by a final extension of 68 °C for 5 minutes. Thin-walled 8-row PCR microtubes (BM Equipment) were used. All PCR amplicons were verified using agarose gel electrophoresis (Figure 1C). The PCR amplicons subjected to ONT library preparation were purified using Ampure XP magnetic beads (Beckman Coulter) and quantified using a Qubit Fluorometric Quantification Assay (Invitrogen).

### 2.3 ONT nanopore sequencing and data analysis

**Figure 2:**
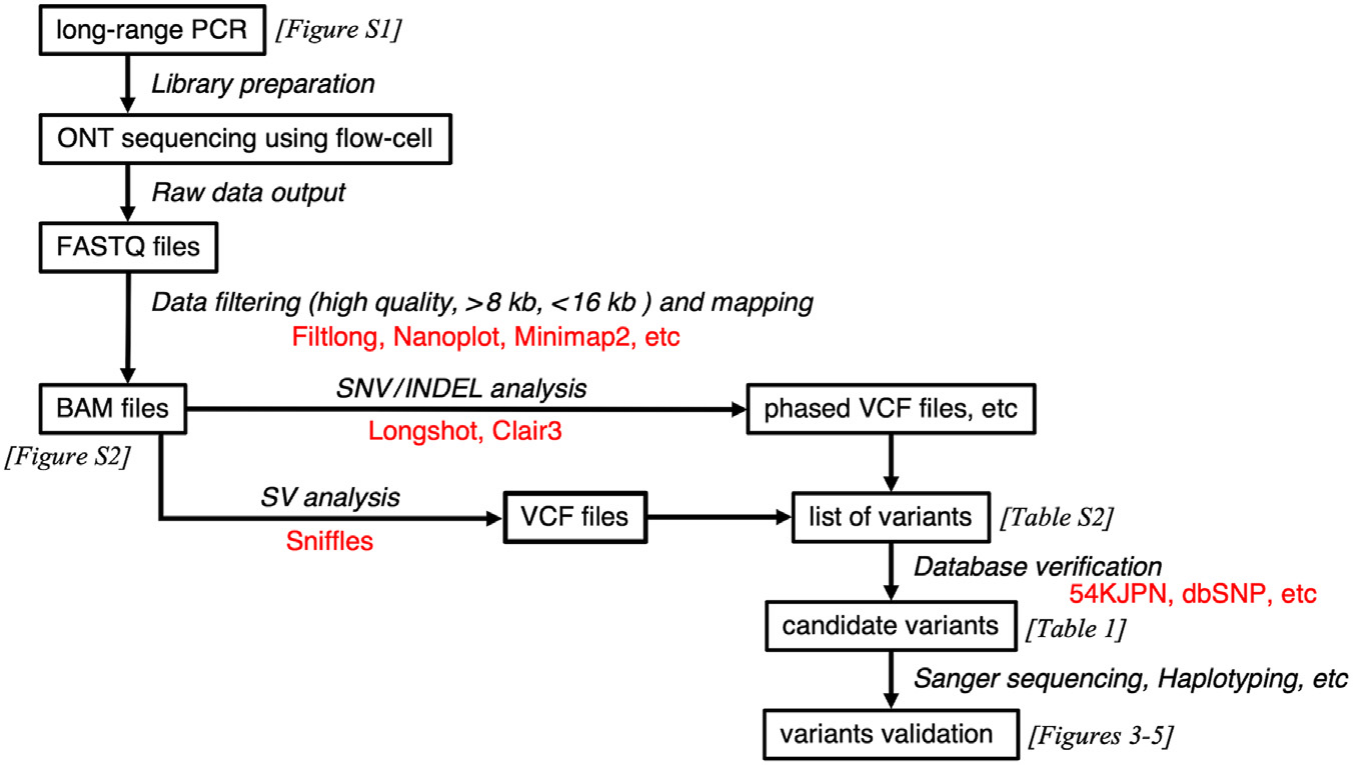
Workflow of ONT nanopore sequencing data analysis in this study. Each step of the workflow is annotated with the corresponding algorithms and databases used, shown in red. BAM, binary alignment map, a comprehensive raw data of genome sequencing; INDEL, insertion–deletion variant; FASTQ, format containing nucleotide sequences and the corresponding quality scores; ONT, Oxford nanopore technology; SNV, single-nucleotide variant; SV, structural variant; VCF, variant call format.

An overview of the analytical workflow is presented in Figure 2. The final 20-fmol DNA library was prepared from long-range PCR amplicons using a ligation kit (SQK-LSK114) according to the manufacturer’s instructions (ONT). ONT nanopore sequencing was performed on a GridION sequencer integrated with MinKNOW v22.08.9 (later upgraded to v22.12.5 and v24.11.8) with a run time of 2 to 12 hours using an R10.4 flow cell. Base calling was performed using Guppy v6.2.11 (subsequently upgraded to v6.4.6) and Dorado v7.6.7 in the high-accuracy model.

Reads with a quality >9 and a length ranging between 8000 and 16,000 nucleotides were selected using Filtlong (github.com/rrwick/Filtlong/) combined with Nanoplot [26]. The selected reads were then mapped using Minimap2 to generate binary alignment map files [27], which were explored using the latest Integrative Genomics Viewer software [28]. The variants were called using Clair3 v0.1-r12 [29] and Longshot v0.4.0 [30]. WhatsHap v1.6 was used for haplotype phasing [31], and Sniffles v2.2 was used for SV calling [32]. The human genome sequence (GRCh38/Hg38) downloaded from the University of California Santa Cruz (UCSC) Genome Browser Database (genome.ucsc.edu/cgi-bin/hgGateway?db=hg38) was used as the reference sequence for data analysis.

The identified variants were named according to the Human Genome Variation Society Nomenclature (hgvs-nomenclature.org/stable/). Allele frequencies and other related details of SNVs and insertion–deletion variants (INDELs) were investigated using the 54KJPN database on jMorp (jmorp.megabank.tohoku.ac.jp), which is an SNV/INDEL allele genotype frequency panel constructed from short-read whole-genome sequencing analyses of approximately 54,000 Japanese individuals. We used dbSNP (www.ncbi.nlm.nih.gov/snp) and ClinVar (www.ncbi.nlm.nih.gov/clinvar) to verify the identified variants and their clinical significance. The Leiden Open Variation Database (https://www.lovd.nl) and Human Gene Mutation Database (https://www.hgmd.cf.ac.uk/ac) were consulted to examine any reported phenotype associations. Rare, nonsynonymous variants with an allele frequency <0.01 in the 54KJPN database were considered candidate pathogenic variants and were validated by Sanger sequencing.

### 2.4 Sanger sequencing

The PCR primer pairs used for Sanger sequencing were designed using Primer-BLAST at the National Institutes of Health to amplify the amplicons covering each variant. PCR was performed in 20 to 50 μL reaction, containing 0.2 to 0.5 μM each of the forward and reverse primer, 1× PrimeSTAR GXL Buffer, 0.2 mM dNTP mix, 50 to 200 ng gDNA or 0.05 ng PCR amplicon, and 0.5 to 1.25 units of PrimeSTAR GXL DNA Polymerase (Takara Bio). The PCR cycling conditions were as follows: initial denaturation of 94 °C for 2 minutes, followed by 30 cycles of 98 °C for 10 seconds, 68 °C for 1 minute, or 30 cycles of 98 °C for 10 seconds, 60 °C for 15 seconds, 68 °C for 3 minutes, followed by a final extension at 68 °C for 5 minutes. The PCR amplicons were verified by agarose gel electrophoresis and treated with an ExoSAP-IT kit (Thermo Fisher Scientific). The amplicons were purified using CleanSEQ (Beckman Coulter) and sequenced in both directions using a Big-Dye Terminator v3.1 Cycle Sequencing Kit (Applied Biosystems) and an Applied Biosystems 3500xL Genetic Analyzer.

### 2.5 Haplotyping

ONT nanopore sequencing reads covering the region of interest were randomly extracted from raw data and manually inspected. A 20-bp specific sequence (gacttcctggttcaagggat located at chr12:6081426-6081445, which is near the end of the PCR21 region and in the middle of the PCR6 region) was used to locate PCR21 reads in the format containing nucleotide sequences and the corresponding quality scores (FASTQ) file containing over 100,000 reads. Only reads with a length of ∼11,941 or 9342 bp and with this 20-bp sequence near the end, either from the forward or reverse direction, were considered to be generated from the PCR21 amplicon and were selected for haplotyping. Sequence data from 50 eligible reads (25 from each of the forward and reverse directions) were imported into Genetyx software (Nihon Server). All 50 reads were divided into normal-length (∼11,941 bp) and deleted-length (∼9342 bp) reads and assembled into the corresponding PCR21 reference sequences (amplicon sequence with or without deletion) using Sequencher software (Gene Codes). Based on the heterozygous SNVs identified in the PCR21 region, nucleotides of each read at each heterozygous SNV position were visually checked using Sequencher, and the distribution of heterozygous SNVs per allele was used to distinguish the reads derived from different alleles.

## 3 Results

### 3.1 Amplification of the *VWF* gene by long-range PCR

Long-range PCR was designed to ensure full coverage and accurate amplification of *VWF* (Figure 1B). A gDNA sample from a healthy donor was used to determine optimal PCR conditions. For each PCR, 2 to 4 primer pairs were designed targeting the corresponding ∼15-kb region, and the amplification efficiency and specificity of all primer pair combinations were examined by agarose gel electrophoresis after PCR. The selected primer pairs were used to investigate the minimum amount of template DNA required and the most appropriate cycling condition (2 or 3 steps) for each PCR. After several optimizations, 21 primer pairs were determined (Supplementary Table 1), and 12- to 15-kb 21 PCR amplicons covering the entire *VWF* (184.7 kb from 4 kb upstream to 5 kb downstream of *VWF*) were successfully generated (Figure 1C).

### 3.2 ONT nanopore sequencing

A DNA library was prepared by combining 21 long-range PCR amplicons from healthy donor samples and applied to a flow cell. ONT nanopore sequencing generated 448.01 Mb of passed bases, with 24.89k reads selected using Filtlong. The sequence within the 184.7-kb region encompassing the entire *VWF* gene was successfully read, with ONT coverage ranging from approximately 80 to 9791 reads (Figure 1D). In contrast, coverage in the *VWFP1* pseudogene region (chr22:16,690,097-16,704,461) (Supplementary Figure 3) was below 28 reads, confirming the specificity of the primer pairs used in our long-range PCR. Variant calling was not performed for the healthy donor sample but was conducted for the 3 patient samples (Supplementary Table 2).

### 3.3 Long-range PCR and ONT nanopore sequencing using patient samples

To assess the applicability of this genetic analysis method in VWF-related disorders, we selected and analyzed DNA samples from the only 3 patients registered under the keyword VWD in the NCVC Biobank. These samples were processed using the optimized protocol validated in the healthy donor analysis (Figure 2). Following long-range PCR and agarose gel electrophoresis, some primers with low efficiency—likely due to patient-specific SNP variations—were replaced to improve DNA amplification and ensure optimal ONT nanopore sequencing (Supplementary Figure 1).

ONT nanopore sequencing was successfully performed on the 3 patient samples, yielding 501.25, 483.38, and 250.88 Mb of passed bases, respectively—comparable with the output obtained from the healthy donor. For variant calling, 20,320, 22,550, and 14,250 high-quality reads were selected from each sample based on quality score and read length, as described in the Methods section. Using these reads, we identified intronic variants (245, 234, and 254 SNVs and 59, 60, and 59 INDELs) and exonic variants (10, 12, and 11 SNVs) in the 3 respective samples (Supplementary Table 2 and 3). The read coverage profiles of the patient samples exhibited patterns consistent with those of the healthy control (Supplementary Figures 2 and 3).

Rare, nonsynonymous variants with allele frequencies <0.01 on 54KJPN were considered potentially pathogenic. No candidate variants were detected in 1 patient sample, whereas 1 rare missense SNV NC_000012.12:g.5976222C>A NM_000552.5:c.7326G>T p.(Gln2442His) and 1 rare 2599-bp deletion NC_000012.12:g.6087520_6090118del NM_000552.5:c.657+5342_657+7940del were identified in the other 2 patient samples (Table). The p.(Gln2442His) variant is registered as rs139290955 in dbSNP and has an allele frequency of 0.0049998 in the Japanese population (54KJPN) and 0.000012 in the global population (dbSNP). The g.6087520_6090118del variant was not listed in any of the referenced databases, including dbSNP, Leiden Open Variation Database, and Human Gene Mutation Database.

**Table:** Candidate variants of the *VWF* gene identified in Japanese patient samples.

Patient	Region on *VWF*	Identified variant	AF on 54KJPN	ClinVar	LOVD and HGMD	Clinical information
1	—	—	—	—	—	VWD 1: VWF:Rco 26%, VWF:Ag 39%
2	Exon 43	p.(Gln2442His)	0.0049998	—	Not reported	LVAD-AVWS: no available VWF result
3	Intron 6	g.6087520_6090118del	—	—	Not reported	LVAD-AVWS: no available VWF result

AF, allele frequency; AVWS, acquired von Willebrand syndrome; HGMD, Human Gene Mutation Database; LOVD, Leiden Open Variation Database; LVAD, left ventricular assist device; VWD, von Willebrand disease; VWF, von Willebrand factor.

### 3.4 Validation of pathogenic candidate variants

**Figure 3:**
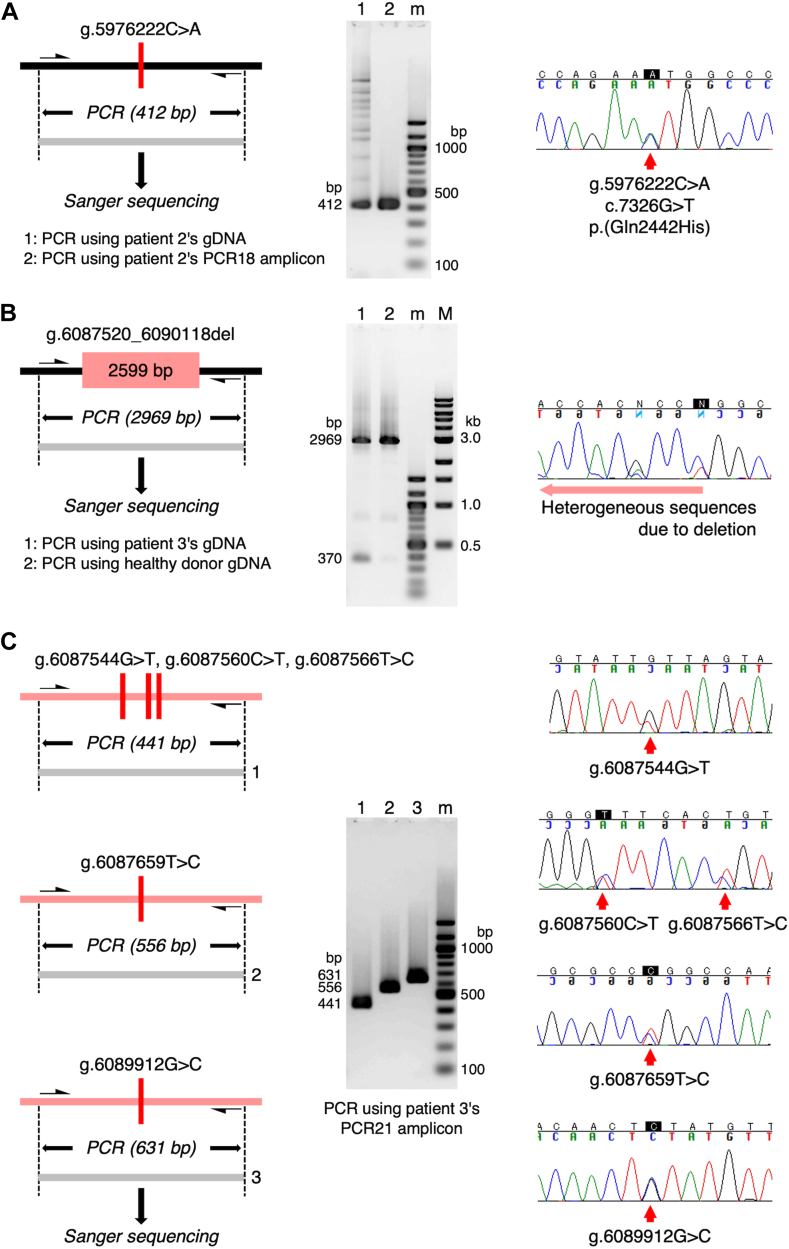
Validation of candidate variants by Sanger sequencing. (A) Confirmation of the heterozygous missense SNV p.(Gln2442His) identified in patient 2. The 412-bp polymerase chain reaction (PCR) products using genomic DNA (gDNA) from patient 2 (lane 1) and using a long-range PCR amplicon from the patient’s gDNA (lane 2) were directly sequenced using the Sanger method. (B) Confirmation of the heterozygous 2599 bp deletion g.6087520_6090118del identified in patient 3. PCR amplification of the region containing the deletion produced the 2969-bp and 370-bp bands using patient 3 gDNA (lane 1), but only the 2969-bp band using healthy donor gDNA (lane 2). Direct Sanger sequencing of the patient’s PCR products indicated the heterogeneous sequences from the deleted site. (C) Identification of heterozygous SNVs within the g.6087520_6090118del region. Three PCR products (441, 556, and 631 bp) using a long-range PCR amplicon from gDNA of patient 3 carries 5 heterozygous SNVs. m, DNA ladders every 100 bp; M, DNA ladders of 0.5, 1, 1.5, 2, 3, 4, 5, 6, 8, and 10 kb. SNV, single-nucleotide variant.

**Figure 4:**
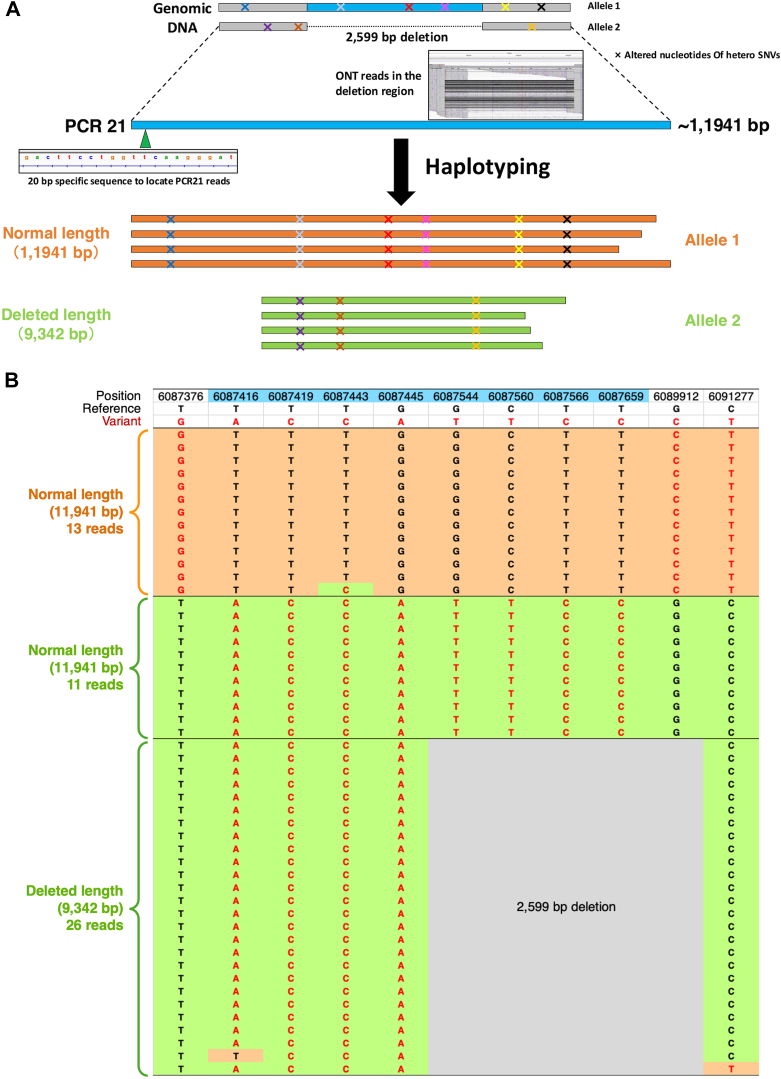
Haplotyping analysis of the polymerase chain reaction (PCR) amplicon carrying g.6087520_6090118del. (A) Schematic representation of the expected haplotyping result of a PCR amplicon with a true 2599-bp deletion under normal circumstances. (B) Fifty reads generated from PCR21 amplicon of patient 3, covering the 2599-bp deletion, were extracted from the raw ONT data. All reads were sorted into 3 types according to the heterozygous SNVs identified in this region and their length. The haplotype of 26 deleted reads (9342 bp) matches that of 11 normal-length reads (11,941 bp). Heterozygous SNVs identified in patient 3 but not in the healthy donor or patients 1 and 2 are shown in light blue. SNV, single-nucleotide variant.

One pathogenic candidate variant, p.(Gln2442His), in patient 2 was confirmed by PCR-direct Sanger sequencing (Figure 3A). The other candidate variant, g.6087520_6090118del, in patient 3 was confirmed in the same manner (Figure 3B). However, the variant calling list generated by Clair3 and Longshot unexpectedly identified 5 heterozygous SNVs in the 2599-bp deletion region of patient 3, which was also confirmed by Sanger sequencing (Figure 3C), indicating that this deletion may be erroneous. Therefore, 50 ONT nanopore sequencing reads generated from the PCR21 amplicon covering this region were visually inspected. The 50 reads could first be divided into 2 length groups: 24 normal reads (average, 11,987 ± 26 bp) and 26 deletion reads (9379 ± 16 bp), subsequently assembled into the corresponding reference sequences (11,941 or 9342 bp). By confirming all heterozygous SNVs in each read, we found that 24 reads of normal length comprised 13 reads from 1 allele and 11 reads from the other allele (Figure 4). Notably, all 26 deleted reads were from the same allele as the 11 normal reads. These results indicated the presence of 3 alleles in this region.

**Figure 5:**
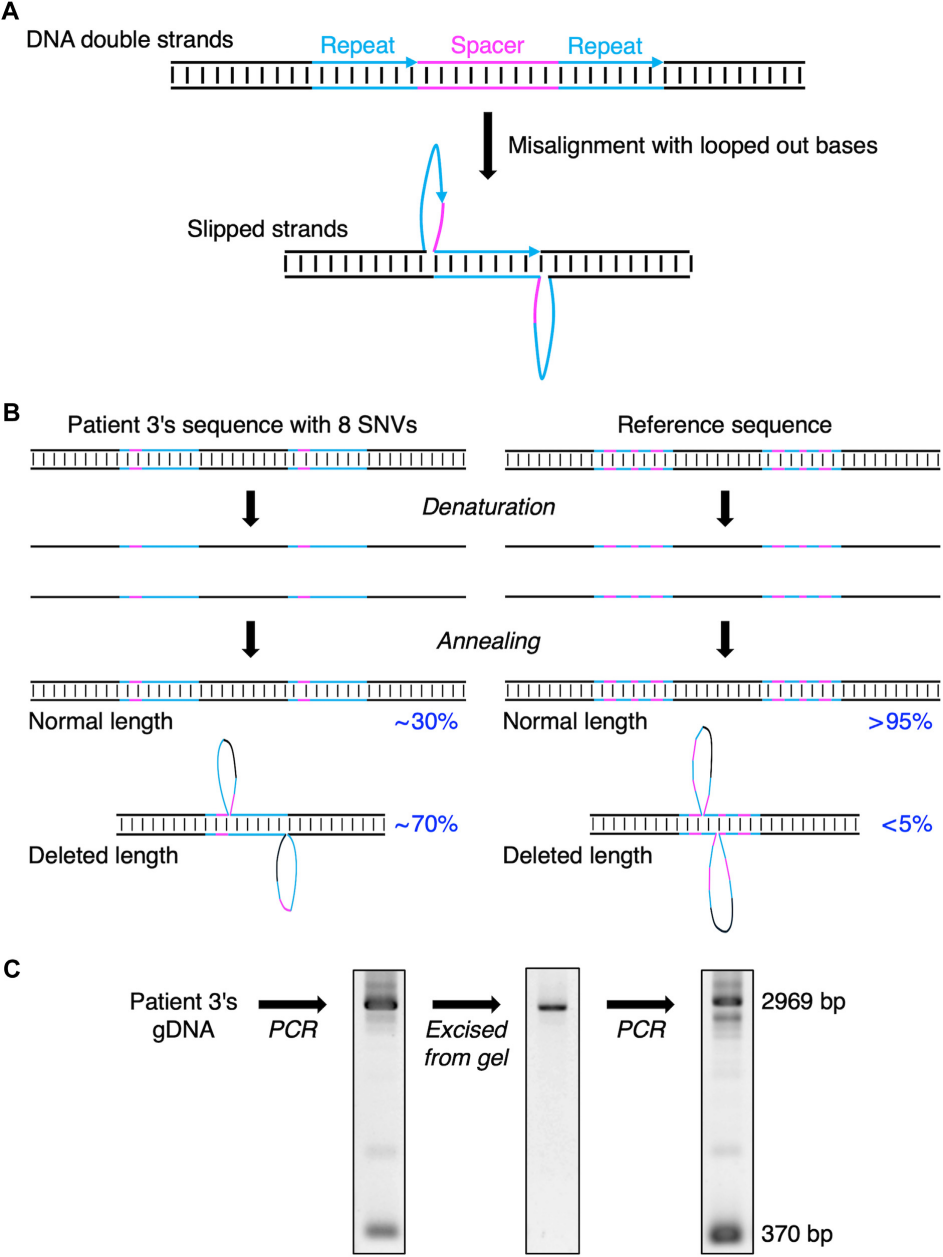
Polymerase chain reaction (PCR) artifacts due to slipped-strand structure. (A) Specific nucleotide sequences with 2 copies of repeats and 1 copy of a nonrepetitive spacer form a slipped-strand structure. (B) Eight SNVs in the g.6087416-6087659 region on 1 allele of patient 3 caused a slipped-strand structure, which tended to produce more deletion amplicons in long-range PCR (∼70% of total amplicons). In contrast, alleles with reference sequences in the region are less likely to have a slipped-strand structure due to incomplete repeat sequences, resulting in few deletion reads (<5% of total amplicons). (C) The first PCR was performed with gDNA from patient 3 as shown in Figure 3B. The second PCR was performed with the same primers using the normal-length band (2969 bp) excised from the agarose gel as a template. The second PCR also produced a 370-bp band, indicating that this phenomenon was a PCR artifact. gDNA, genomic DNA; SNV, single-nucleotide variant.

To investigate the factors underlying this confusing result, we analyzed the flanking nucleotide sequence around the 2 breakpoints of the 2599-bp deletion variant g.6087520_6090118del and considered it an artifact of long-range PCR due to a special sequence called “direct repeats” [33]. It can form a slipped-strand structure, a type of mispairing caused by hairpin loops between 2 highly repetitive sequences (Figure 5A). Although this type of sequence also exists in the *VWF* reference sequence, it is short and appears to have little effect on long-range PCR. Accordingly, we examined the raw ONT data from the healthy donor and patients 1 and 2 and found that deletion reads were remarkedly rare in these 3 samples (Supplementary Figure 4). In patient 3, 8 heterozygous SNVs in the region of g.6087416-6087659 on 1 allele (Figure 4) lengthened the repeat unit and easily generated more deletion amplicons than the reference sequence (∼70% vs <5%) (Figure 5B). Long-range PCR using the excised 2969-bp band as a template generated the original 2969-bp band, along with a short 370-bp band, indicating a correlation between the occurrence of the artifact and the specific sequence during long-range PCR (Figure 5C).

## 4 Discussion

In this study, we present a genetic analysis method using ONT nanopore sequencing to rapidly identify variants in *VWF* with minimal interference from multiple repetitive sequences or the pseudogene *VWFP1*. To develop a practical and feasible approach using the advantages of ONT, we used long-range PCR to amplify the entire *VWF* gene sequence, which can provide PCR products favorable for subsequent library preparation and long-read sequencing.

Although long-range PCR is widely used in NGS workflows [34] and is considered a cost-effective option, amplifying ∼15-kb products from the 178-kb *VWF* remained technically challenging in our study. Through extensive optimization—focusing on DNA polymerase choice, primer design, template quantity, and PCR microtube selection—we established the most effective protocol, as described in the Methods section. The specificity of the resulting PCR amplicons was subsequently validated by ONT nanopore sequencing. Notably, we found that using thin-walled PCR microtubes significantly improved amplification success and yield for ∼15-kb fragments, and we recommend this as a practical tip for similar long-range PCR applications.

Due to the high sequence homology between *VWF* and its pseudogene *VWFP1,* previous studies have reported that short-read NGS technologies fail to adequately cover exon 26, as they cannot reliably map reads to this region [35]. Our data showed no significant drop in coverage in exon 26 compared with its neighboring exons 25 and 27 (Figure 1D and Supplementary Figures 2), further highlighting the strength of ONT for analyzing structurally complex genomic regions such as *VWF*.

Our study demonstrated the long-range PCR, especially when combined with ONT sequencing, serves as a reliable and cost-effective tool for genomic analysis, consistent with previous reports [36,37]. However, the detection of PCR artifacts in the amplicons from patient 3 highlights a limitation of this approach: the amplification of regions harboring repetitive sequences remains technically challenging. In this case, the presence of “direct repeat” sequences in *VWF* is presumed to have led to the formation of secondary structures, such as hairpin loops, which interfered with accurate amplification [38,39]. Therefore, rigorous validation and confirmation of variants, particularly those located within repetitive or structurally complex regions, are required to avoid false-positive or misleading results.

The first patient enrolled in our study was diagnosed with type 1 VWD, as shown in Table. Genetic defects associated with this type of VWD are reportedly distributed throughout the *VWF* gene. However, due to the high polymorphism of *VWF*, rare variants do not always cause the disease [40]. Additionally, not all patients with type 1 VWD exhibit a genetic defect in *VWF*, with reported variant detection rates ranging from 45% to 68% [41,42]. This may explain why no candidate variant was identified in this patient.

The second and third patients were also registered under the keyword VWD in the NCVC Biobank. However, after their samples had already been used for genetic analysis, we were informed that both had actually been diagnosed with left ventricular assist device–associated acquired von Willebrand syndrome, not VWD, according to their clinical records (Table). Nevertheless, a p.(Gln2442His) variant located in the C3 domain of VWF was identified in the second patient. Using 2 *in silico* approaches, this variant was previously predicted to be a deleterious mutation in an Asian population [43]. Notably, this is the first time the variant has been identified in a clinical case, suggesting a potential association with acquired von Willebrand syndrome risk. To explore its impact on VWF structure and function, we conducted further investigations. However, there was no significant correlation with synthesis, secretion, or ADAMTS13 cleavage (data not shown). Given that the mutation is located near the binding site of platelet receptor glycoprotein (GP)IIb/IIIa, it is possible that the mutant rVWF exhibits impaired GPIIb/IIIa-binding affinity. Applying the cell-based GPIIb/IIIa-binding assay reported in 2019 [44] may be a suitable approach to further assess this potential effect.

This study has several limitations. First, we analyzed only 3 patient samples, including just 1 VWD case. Nevertheless, leveraging the strengths of ONT sequencing, we detected a substantial number of variants in intronic regions, with >290 per sample, which was >20-fold, greater than the number of exonic variants (Supplementary Table 3). Although the pathogenic significance of intronic variants in *VWF* remains underexplored, emerging evidence suggests their potential relevance. For instance, in 2022, a homozygous deep intronic variant (c.997+118T>G in intron 8) was identified in a patient with type 3 VWD and classified as pathogenic due to the creation of a novel donor splice site, resulting in aberrant mRNA with a premature stop codon [45]. Additionally, a *VWF* SNP and haplotype analysis from the Atherosclerosis Risk in Communities cohort identified 18 SNPs associated with VWF levels in 7856 European-descent subjects in 2011 [46]. These findings highlight the potential role of intronic variants in VWD pathogenesis. Given this, we used splicing prediction bioinformatics tools—Neural Network and the Berkeley Drosophila Genome Project—to assess the impact of 7 intronic variants with an allele frequency below 0.01 in our VWD case. While no novel donor or acceptor splice sites were predicted (data not shown), expanding this approach through collaboration with a larger VWD registry could help identify previously unrecognized disease-causing intronic variants, further elucidating the mechanisms of VWD. Second, the algorithms (Clair3 and Longshot) used for variant calling and haplotype phasing were not designed for long-range PCR-based ONT nanopore sequencing analysis. Accordingly, we are developing a novel software with lower error rates. Upon application to *VWF* analysis, it may detect variants that are overlooked by the current software.

In conclusion, we developed a novel method for *VWF* genetic analysis by combining long-range PCR with ONT nanopore sequencing. This approach provides a powerful tool for investigating the pathogenetic mechanisms of VWF-related disorders and holds the potential to serve as a first-line sequencing method as long-read sequencing technology becomes more widely adopted in the future.
