# Supplementary material for: Genetic analysis using long-read sequencing to overcome the difficulties in VWF gene
Source: Res Pract Thromb Haemost. 2025 May 17;9(4):102888. doi: 10.1016/j.rpth.2025.102888 (PMC12173654; doi:10.1016/j.rpth.2025.102888)
Supplement: Supplementary Figures 1-4 [file mmc1.pdf]

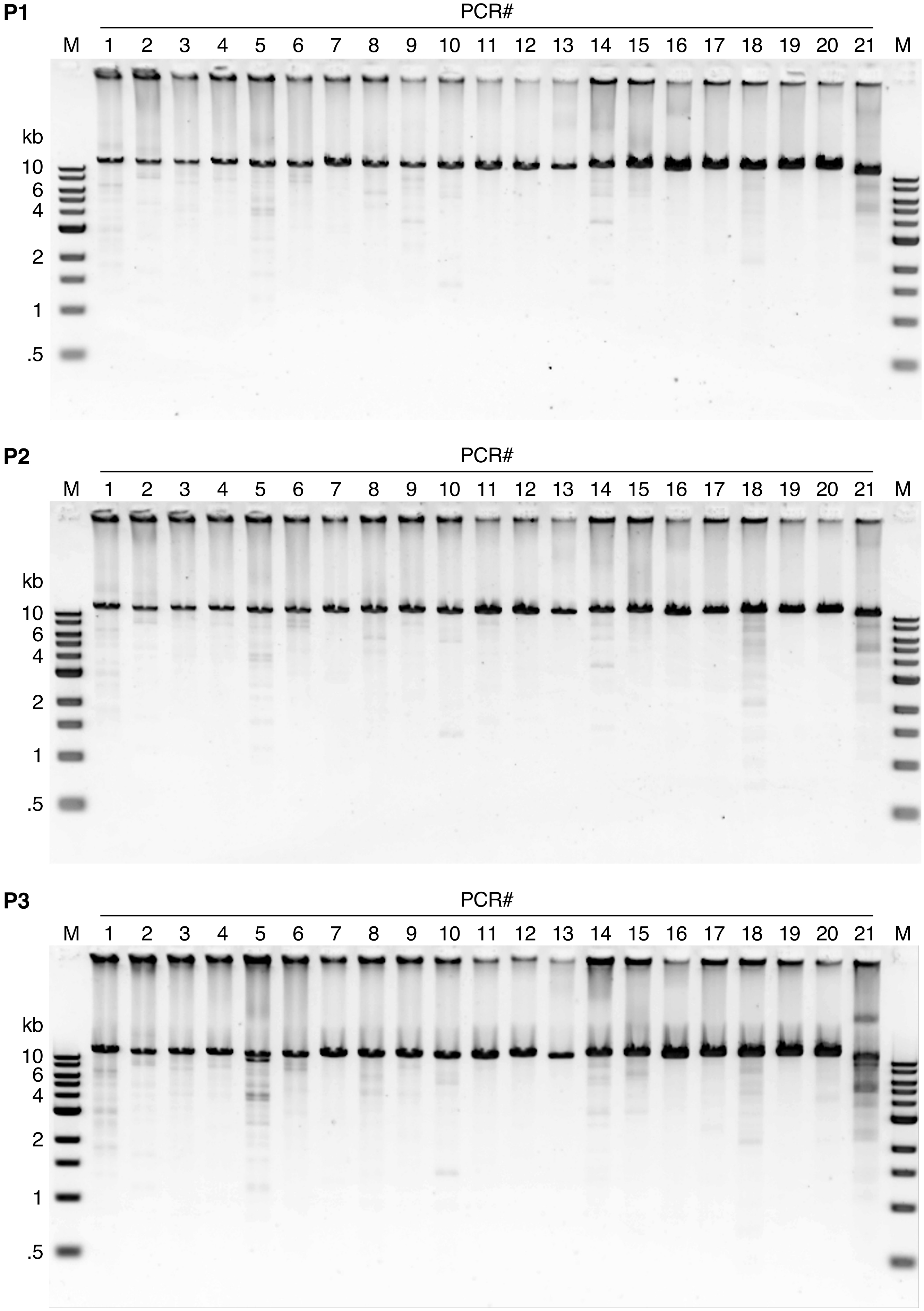

Supplementary Figure S1. Long-range PCR of three patient samples.
Representative 1.2% agarose gel electrophoresis images of PCR amplicons generated from DNA samples of patient 1 (P1), patient 2 (P2), and patient 3 (P3). M, DNA ladder of 0.5, 1, 1.5, 2, 3, 4, 5, 6, 8, and 10 kb.

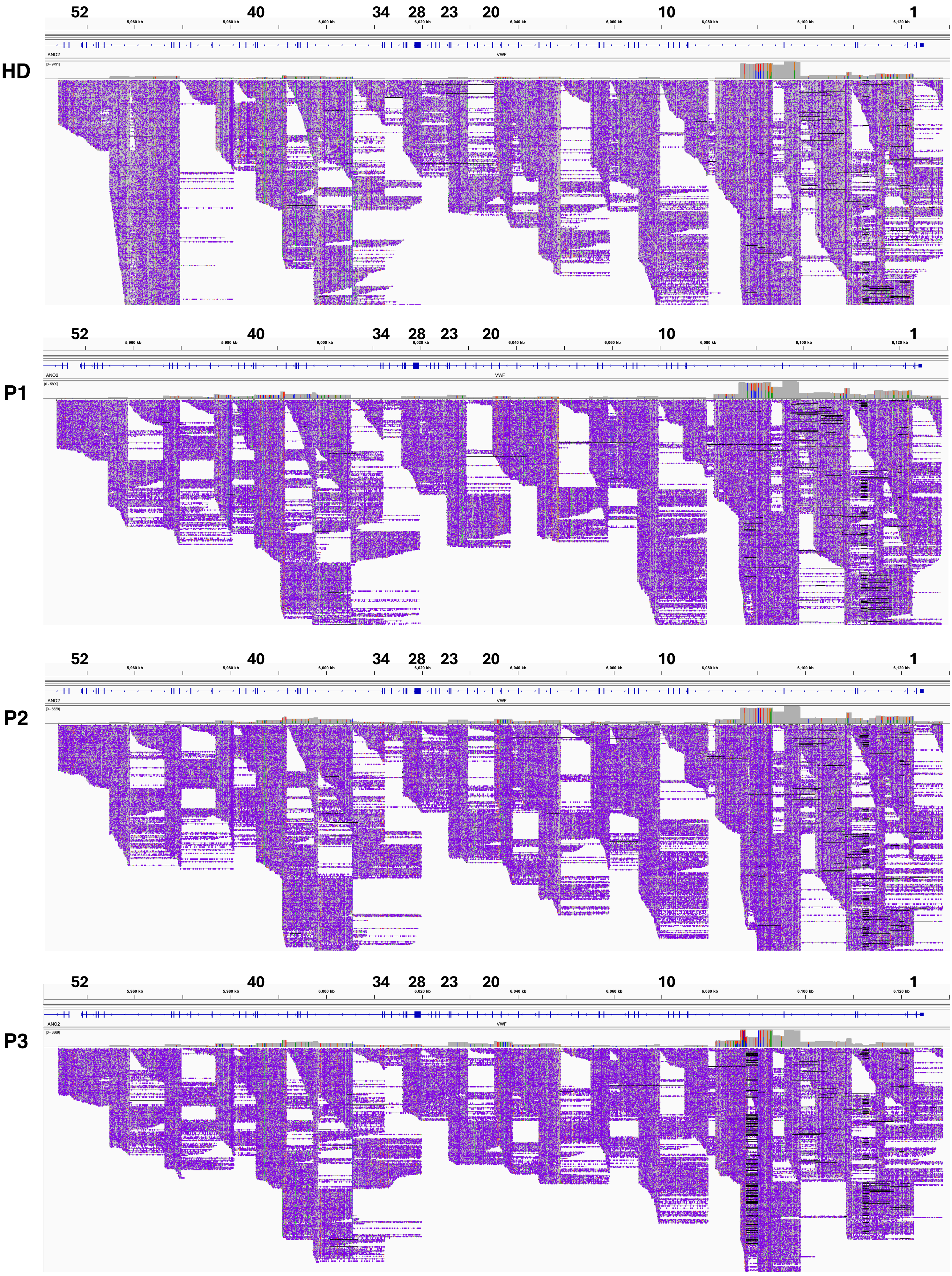

Supplementary Figure S2. ONT nanopore sequencing reads across the entire *VWF* gene region. Long-read sequencing data from a healthy donor (HD), patient 1 (P1), patient 2 (P2), and patient 3 (P3) aligned using IGV software. The coverage height represents the number of reads at each nucleotide position. The numbers indicate the locations of *VWF* exons.

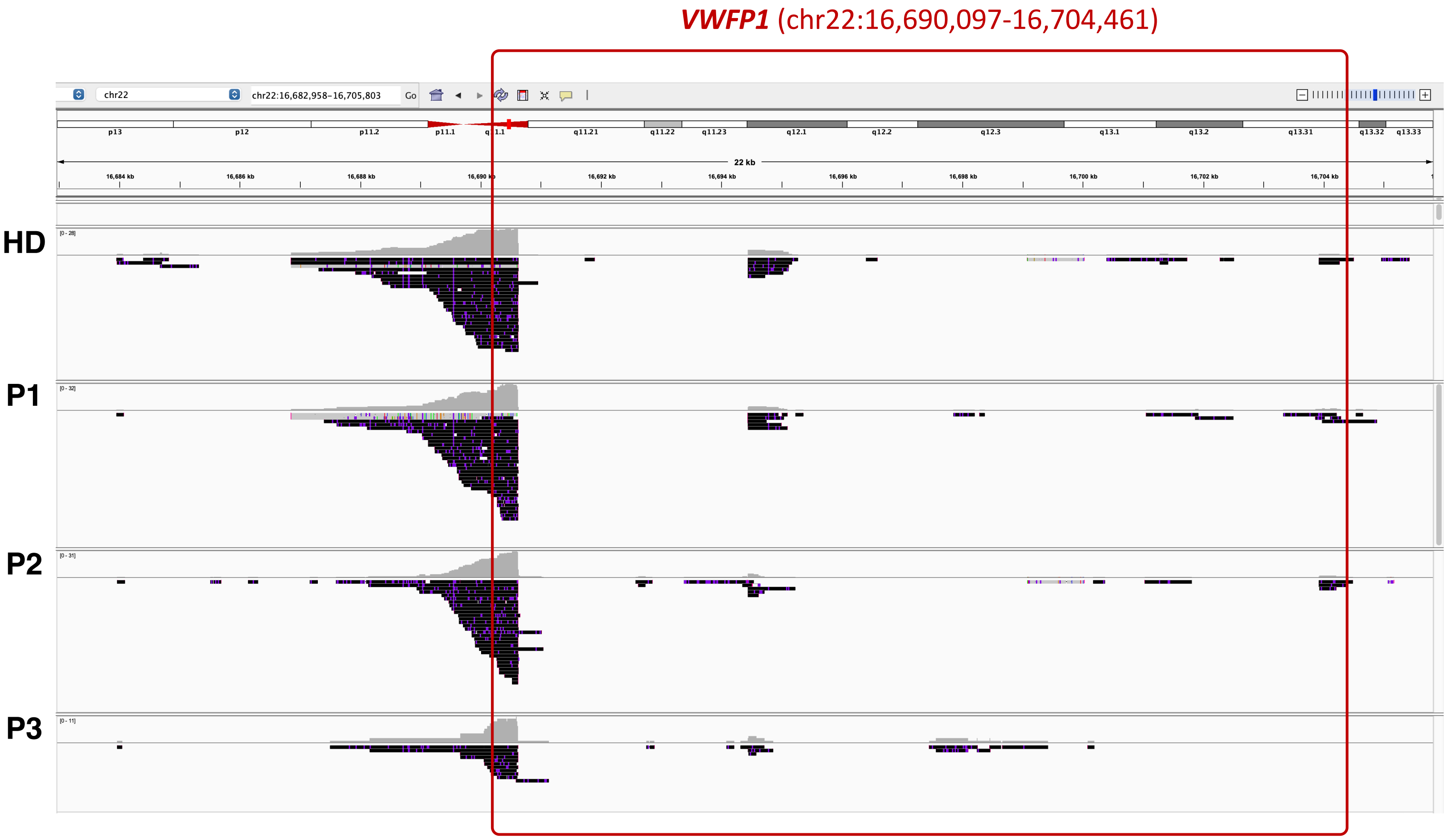

Supplementary Figure S3. ONT nanopore sequencing reads in the *VWFP1* pseudogene region. Long-read sequencing data from a healthy donor (HD), patient 1 (P1), patient 2 (P2), and patient 3 (P3) aligned using IGV software. The coverage height represents the number of reads at each nucleotide position. The red box highlights the *VWFP1* pseudogene region.

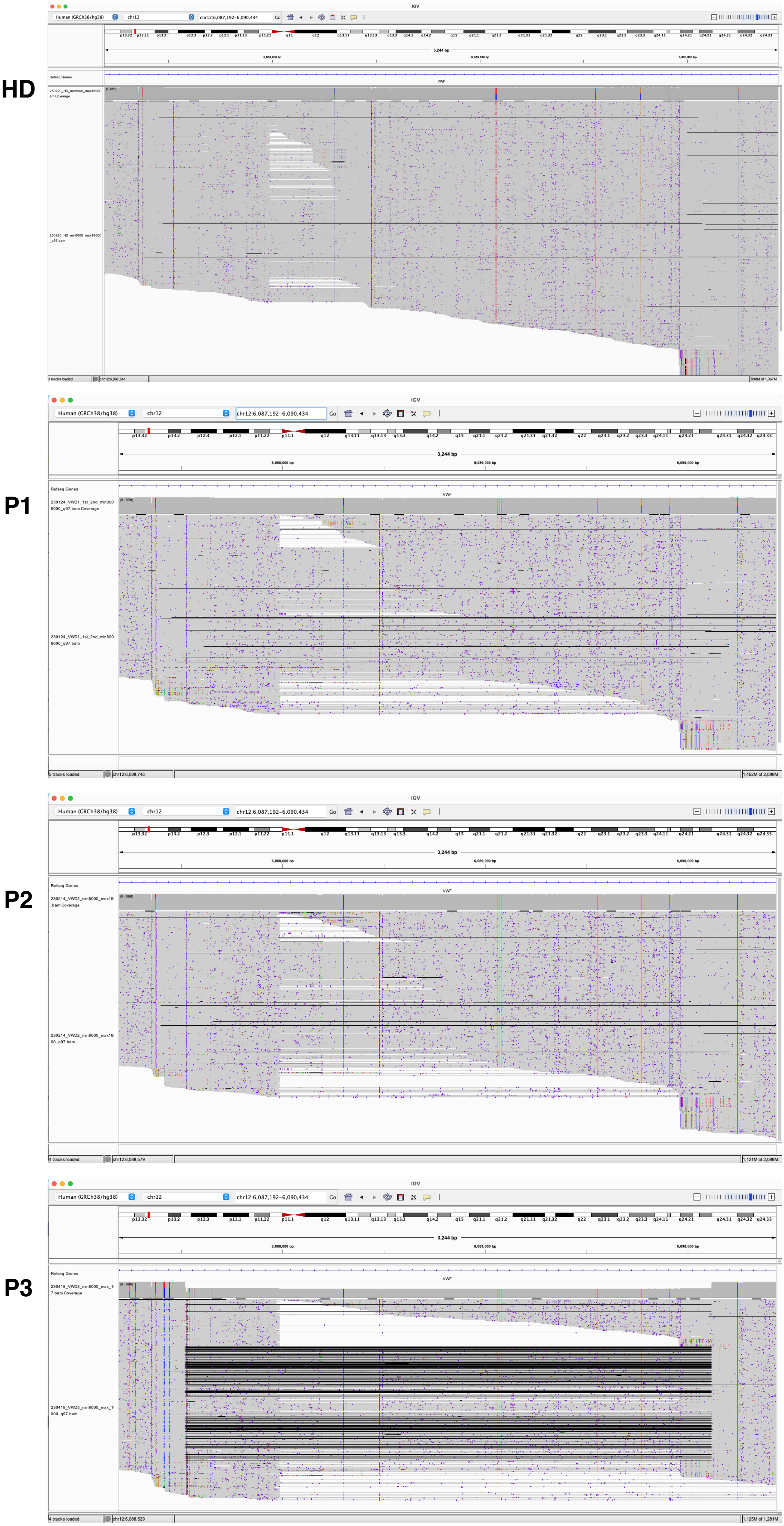

Supplementary Figure S4. ONT nanopore sequencing reads in the g.6087520_6090118del region. Long-read sequencing data from a healthy donor (HD), patient 1 (P1), patient 2 (P2), and patient 3 (P3) aligned using IGV software. Black horizontal lines represent deletion reads.
