# Supplementary material for: Genetic analysis using long-read sequencing to overcome the difficulties in VWF gene
Source: Res Pract Thromb Haemost. 2025 May 17;9(4):102888. doi: 10.1016/j.rpth.2025.102888 (PMC12173654; doi:10.1016/j.rpth.2025.102888)
Supplement: Supplementary Tables 1-3 [file mmc2.pdf]

Supplementary Table S1. Primer pairs for *VWF* long-range PCR.

PCR#	Forward primer	Reverse primer	Product size (bp)	Template DNA (ng)	Amplication cycle
1	CCCTCTAACCCCATCTCTCCGTTTCT	GAGACCAGCATCAGGAAGTCCAAGG	13929	>200	2-step
2	GACCTTGGGCGCTTTCCACTTAGAT	GGTAGAGTCCTGCACCAATCAGAGAAT	14088	>100	2-step
3	CCTGCCAAGAGAGAGCCATCAACAA	TCTGCATGTGCTCAGAAAGGGAAGG	14527	>100	2-step
4	ATGTTCCCTTGCCAAAGCTAAACAC	GGTCCCTGTGGAGGAAAGTTTCAGG	14015	>100	2-step
5	GGGGTCTCAGGATCTCCTTCCTACA	GATCCTGCATTCCAAGGGTCCTTCC	12516	>100	2-step
6	TCTCTGTGACCATCATCTCCTCGCT	TGGGGACAGTGACCATAACTAGGCA	13208	>200	2-step
7	ACAAGACGCCAACTCCTCTTTGGAC	CTGGCAGATCCCACTGAAGGTGAAG	14457	>100	2-step
8	CACCCACGCACACAAGCATATTTCC	TTTCAGTGGGCCCTTAGCATTCCAG	14412	>200	2-step
9	TCTATGGGCCTGGGACCTTCTACTG	CACTCCAGGTCATAGTTCTGGCACG	14770	>200	2-step
10	GTGCTCAGAAAGGGAAGGGCTGATT	AAGGGGGAAACTGAGGCAAAGAAGG	13813	>200	2-step
11	TTTGTCTCCTCTCCAGCTCTCTTAT	ACTCTGTGTCCATACCACCA	13314	>200	2-step
12	CTTCATCCTCCTGGATTTCTTGCT	ACCAGGACAGAGGTTGGTAT	13573	>200	2-step
13	TGGGAATATGGAAGTCATTG	ACGAATAAGATTAAAACCCAATGGAAAGT<br>AAACCCAATGGAAAGTAGAAATAATGAA‡	14398<br>14385	>200	3-step
14	TGCAGGATTTGTTAGGATTT	AAGGCAAGTTAGTGAGAATGGCTCT<br>CTAAAAATCCCCTATGTCCTTGAGC§	14744<br>14935	>200	3-step
15	ACTTTCCATTGGGTTTTAATCTTATTCGT	ATTTTAGGCCAAGACGGAGGAGTCT	14666	>200	3-step
16	CCACTTTGGCTACCTGGACGTCTTT	GGGGACTCTTAAGTCTGACCGTTGC	12872	>100	2-step
17	AGCTCTTGGTTCAAATCCTGCGTCT	CCACAGACAATGCTCGGACATCCTT	14771	>100	2-step
18	CGGGAACAAGTCTAGGAGGCTACAG	CCTGACTGCCCTGATTGGACTTCAG<br>AGCCCAGATGTGTTCTAGGAGGGTT†	14517<br>14429	>200	2-step
19	ATGAGTGTGTCCGAGTGAAGGAGGA<br>CTGGGTGCCTCAGTCAGGTGATTTT†	AAGGAGCCCAGAGACAAAACGACAG	14630<br>14896	>100	2-step
20	TCTAAGCTTGGCAACAATGGACCAA	AGCAGAAGCAGTTGCTGGAAAGGAT	14837	>100	2-step
21	ACCACCTTCCTGCCCTTATCCAGAG	AACACAGCTCTCCTCAGACAAAGGC<br>ACACCTCCAAAGAACACAGCTCTCC†	11929<br>11941	>300	2-step

†Used only for the three patient samples. ‡Used only for patient 2 and patient 3 samples. §Used only for the patient 3 sample.

Supplementary Table S2. ONT nanopore sequencing data and variant calling results of *VWF*.

DNA samples	Run time (h)	Bases called (Mb)		Reads selected by Filtlong		Variants called by Clair3†		Variants called by Longshot‡
		Pass	Fail	Number of reads	Mean read length (bp)			
HD	2	448.01	365.77	24,892	12,607.7	-	-	-
P1	12	501.25	541.26	20,320	12,583.1	231	59	235
P2	12	483.38	533.85	22,551	12,631.4	193	60	210
P3	5	250.88	490.41	14,245	12,110.6	242	59	239

†Clair3 is a symphonizing pileup and full alignment tool for high-performance long-read variant calling, including single-nucleotide variants (SNVs) and insertion-deletion variant (INDELs).

‡Longshot is a variant calling tool for diploid genomes and SNVs.

Pass: Number of bases called from reads ranging from 5 kb to 18 kb.

Fail: Number of bases called from reads shorter than 5 kb or longer than 18 kb.

HD, healthy donor; P1, patient 1; P2, patient 2; P3, patient 3.

Supplementary Table S3. Summary of *VWF* gene variants identified in patient samples.

DNA samples	SNVs called by Clair3† and Longshot‡				INDELs called by Clair3†			
	Intron		Exon		Intron		Exon	
	AF ≥ 0.01	AF < 0.01	AF ≥ 0.01	AF < 0.01	AF ≥ 0.01	AF < 0.01	AF ≥ 0.01	AF < 0.01
P1	238	7	10	0	43	16	0	0
P2	230	4	11	1	40	20	0	0
P3	243	11	8	3*	42	17	0	0

†Clair3 is a symphonizing pileup and full alignment tool for high-performance long-read variant calling, including single-nucleotide variants (SNVs) and insertion-deletion variants (INDELs).
‡Longshot is a variant calling tool for diploid genomes and SNVs.
*All three rare exonic SNVs (AF < 0.01) identified in patient 3 were synonymous variants.
P1, patient 1; P2, patient 2; P3, patient 3.
